# An Empirical Approach for Quantifying Loop-Mediated Isothermal Amplification (LAMP) Using *Escherichia coli* as a Model System

**DOI:** 10.1371/journal.pone.0100596

**Published:** 2014-06-30

**Authors:** Sowmya Subramanian, Romel D. Gomez

**Affiliations:** Department of Electrical & Computer Engineering, University of Maryland, College Park, Maryland, United States of America; Faculty of Biochemistry Biophysics and Biotechnology, Jagiellonian University, Poland

## Abstract

Loop mediated isothermal amplification (LAMP) is a highly efficient, selective and rapid DNA amplification technique for genetic screening of pathogens. However, despite its popularity, there is yet no mathematical model to quantify the outcome and no well-defined metric for comparing results that are available. LAMP is intrinsically complex and involves multiple pathways for gene replication, making fundamental modelling nearly intractable. To circumvent this difficulty, an alternate, empirical model is introduced that will allow one to extract a set of parameters from the concentration versus time curves. A simple recipe to deduce the time to positive, *T_p_* - a parameter analogous to the threshold cycling time in polymerase chain reaction (PCR), is also provided. These parameters can be regarded as objective and unambiguous indicators of LAMP amplification. The model is exemplified on *Escherichia coli* strains by using the two gene fragments responsible for vero-toxin (VT) production and tested against VT-producing (O157 and O45) and non-VT producing (DH5 alpha) strains. Selective amplification of appropriate target sequences was made using well established LAMP primers and protocols, and the concentrations of the amplicons were measured using a Qubit 2.0 fluorometer at specific intervals of time. The data is fitted to a generalized logistic function. Apart from providing precise screening indicators, representing the data with a small set of numbers offers significant advantages. It facilitates comparisons of LAMP reactions independently of the sampling technique. It also eliminates subjectivity in interpretation, simplifies data analysis, and allows easy data archival, retrieval and statistical analysis for large sample populations. To our knowledge this work represents a first attempt to quantitatively model LAMP and offer a standard method that could pave the way towards high throughput automated screening.

## Introduction

Pathogen screening using loop mediated isothermal amplification (LAMP) is growing in popularity because of its practicality, speed and usefulness in laboratories and clinical settings [1], [2]. It compares quite favorably in both sensitivity and selectivity with polymerase chain reaction (PCR) [2], [3], as improvements of the method continue at a brisk pace. Since its introduction [4], methods to quantify LAMP have been implemented. These involve novel real-time sensing techniques utilizing fluorescence [5], turbidity [6] or electrochemical processes [7] and ion-sensitive field effect transistors [8], [9] similar to those used in PCR. Yet, while there is progress in quantitative acquisition of data, a mathematical model to characterize the data remains elusive. Unlike PCR, LAMP is isothermal and does not have cycling steps for counting the number of times the concentration doubles. LAMP is a two-step process that involves the formation of so-called dumbbell structures that become the starting template for the subsequent replication. These structures replicate in an auto-cycling process, which also generate by-products that further amplify the target gene by concatenation of its sequence into progressively lengthening DNA strands [10]. This process is highly parallel, thus making it very difficult to model using first principles. Nevertheless, quantifying LAMP has potentially very important benefits. It could allow unambiguous comparisons between strains, better assessment of probe effectiveness, and better understanding of the effect of sample extraction and purification, and perhaps even allow precise determination of the absolute numbers of gene copies. In this paper, a simple protocol is presented for measuring and characterizing the concentration as a function of time. For illustrative purposes, the technique was demonstrated for detecting vero-toxin producing *Escherichia coli (E. coli)*. The fluorescence intensity of the sample was measured by using a Qubit 2.0 fluorometer at specific intervals of time to provide a time series snapshot of the LAMP amplification process. The intensity was converted into concentration and its behavior with time was fitted using generalized logistics function. This best fit yielded several parameters including time to positive, *T_p_*, the analog of cycling threshold, *C_T_*, in PCR. Those parameters were analyzed for two gene sequences, vero-toxin 1 (VT1) and vero-toxin 2 (VT2), specifically testing LAMP on VT-producing O157 and O45 strains as well as the non-VT producing DH5 alpha strain as negative control. The effect of the starting target concentration as well as influence of the purity of the target DNA was similarly studied.

Vero-toxin (VT) producing *E. coli* was chosen as a test case because of its relative familiarity [11] and because of its culpability in disrupting our food supply. Additionally, as with most food borne pathogens, there is a need for automated screening [12], [13], for which this method can be very useful. This contribution is built upon the work of others [14],[15] who first demonstrated the use of LAMP in rapidly detecting certain VT-producing strains of *E. coli*. Their protocols and primer designs were mimicked though out this project [14].

## Materials and Methods

The primers used are listed in the Tables 1 and 2. The additional loop primers designated as Loop F, Loop F1 and Loop F2 help in accelerating the LAMP reaction and in increasing the specificity of the reaction [16].

**Table 1:** VT1 primer sequence from 5′ to 3′. GenBank database accession no. BA000007.

FIP	GCTCTTGCCACAGACTGCACATTCGTTGACTACTTCTTATCTGG
BIP	CTGTGACAGCTGAAGCTTTACGCGAAATCCCCTCTGAATTTGCC
F3	GCTATACCACGTTACAGCGTG
B3	ACTACTCAACCTTCCCCAGTTC
Loop F	AGGTTCCGCTATGCGACATTAAAT

**Table 2:** VT2 primer sequence from 5′ to 3′. GenBank database accession AE005174.

FIP	GCTCTTGATGCATCTCTGGTACACTCACTGGTTTCATCATATCTGG
BIP	CTGTCACAGCAGAAGCCTTACGGACGAAATTCTCCCTGTATCTGCC
F3	CAGTTATACCACTCTGCAACGTG
B3	CTGATTCGCCGCCAGTTC
Loop F1	TGTATTACCACTGAACTCCATTAACG
Loop F2	GGCATTTCCACTAAACTCCATTAACG

### Sample Preparation

The DNA was extracted from the different strains of *E. coli* cells using two methods: 1. Using the UltraClean Microbial DNA Isolation Kit (MO BIO Laboratories, Inc.) designated as ‘highly purified’, and 2. Using the InstaGene kit, designated as ‘less purified’. The DNA products extracted using either of the extraction kits were diluted to their starting concentrations using 1 mM TE buffer (pH 8.0). The starting concentrations of the different strains of *E. coli* are listed in Table 3.

**Table 3:** Starting concentrations of template DNA used in LAMP reaction.

*E. coli* Strain	Concentration (pg-µl^−1^)
O157	7.54
O45	13.8
DH5	8.47

### LAMP Protocol

The LAMP reaction was carried out in a 25 µl reaction mixture. The LAMP cocktail for both VT1 and VT2, containing 1.6 µM each of FIP and BIP, 0.2 µM each of F3 and B3, 0.8 µM each of the loop primers, 400 µM of each dNTP, 1 M betaine, 20 mM Tris-HCl (pH 8.8), 10 mM KCl, 10 mM (NH_4_)_2_SO_4_, 4 mM MgSO_4_, 0.1% Triton X-100 and 16U Bst DNA polymerase was prepared (24 µl per reaction) [10], [14]. 1 µl of target DNA of concentration in the range of a 1–10 pg/µl is added to the LAMP cocktail. The reaction is allowed to proceed for 60–180 minutes at 65°C.

LAMP reaction was performed in multiple tubes, all containing the same LAMP cocktail with the target DNA of interest (strain O157, O45 or DH5 alpha). A new tube was placed in the heating element set at 65°C every minute for the first 15 minutes and every 15 minutes thereafter for a total time of 2 hours. Incidentally, the heating instrument is homemade and uses aluminum blocks with wells that conform to the shape of 0.2 ml PCR tubes. The temperature variation was less than 0.2°C over the entire course of the experiment. The tubes were then inspected using Qubit 2.0 fluorometer, using Qubit proprietary reagents and dyes. This procedure allowed us to measure the DNA concentration after specific hybridization time.

### Qubit 2.0 Intensity-DNA Concentration Calibration

The Qubit 2.0 protocol for calibrating the fluorescent intensity was followed as described in the manual [www.invitrogen.com/qubit]. Two DNA assay kits were prepared from the calibration standards provided by the manufacturer and their fluorescence signals were measured. An example of the calibration is shown in Figure S1, where the two points corresponding to 0 and 5 ng/µL DNA concentration and their corresponding fluorescence curves are plotted. Since the concentration levels in these experiments in the range of 10–200 ng/µL, the DNA samples were further diluted in order to put them within the range of the calibration. The dilution factor is used by Qubit 2.0 algorithm to calculate the actual concentration. After the calibration routine, the device simply displays the concentration and information about the raw fluorescence intensity is omitted.

### Analyses of LAMP products

Turbidity measurements and electrophoresis were performed at the start of the experiments to ensure that the protocols and primers worked. The turbidity level of the LAMP solution was taken as a qualitative assessment of the quantity of the amplicons, while banding in gel electrophoresis in 1.5% agarose gel is used to corroborate the turbidity results. After the preliminary investigations, the concentration of the LAMP products was recorded using the fluorometer. There were several PCR tubes containing various primers, reagents and the DNA target (O157, O45 or DH5 alpha). For the negative control experiment, the tube contained the VT LAMP primers and reagents but was reacted with the DH5 strain, a non-VT producing *E. coli* strain. After a given duration of at 65°C, a set of samples were removed from the isothermal heater, cooled to room temperature and analyzed. This measurement gave the amount of DNA that has been synthesized up to that point in time, with the assumption that amplification is arrested once the sample temperature falls below 60° C.

## Results

**Figure 1:**
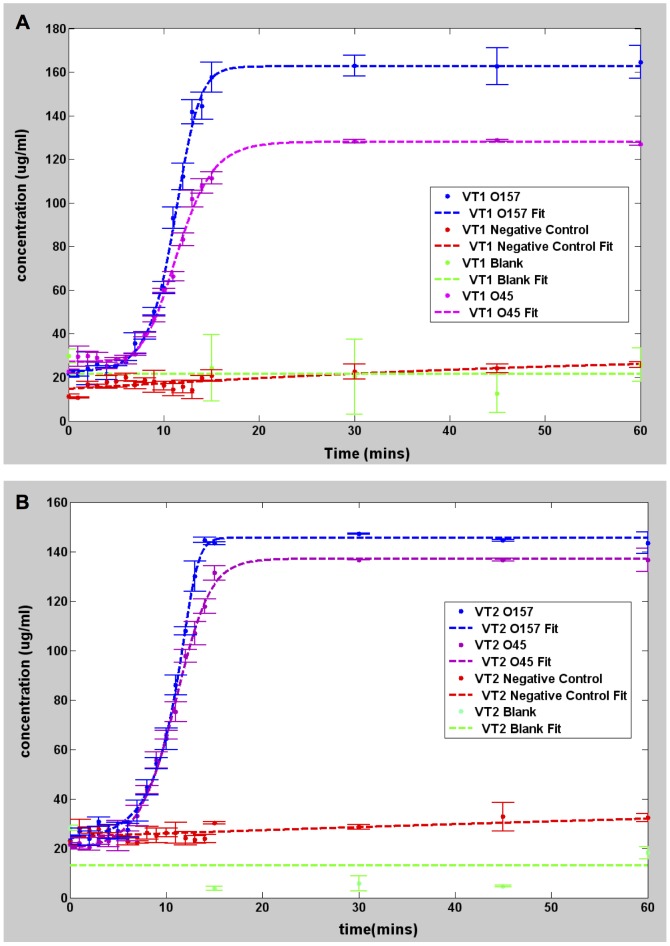
Graphs showing pseudo real time amplification of target DNA. All measurements made using Qubit 2.0. A. Plot showing DNA amplification using *E. coli* O157, 045 and DH5 highly purified genomic DNA as target for VT1 gene. B. Plot showing *E. coli* O157, 045 and DH5 highly purified genomic DNA as targets for VT2 gene.

**Figure 2:**
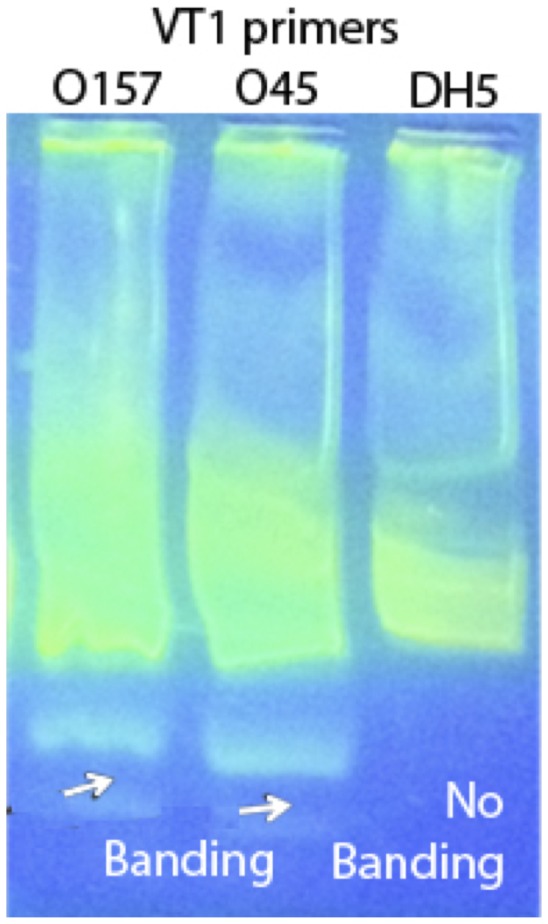
Gel electrophoresis of LAMP products showing bands formed by the positive sample and absent in the negative control.

**Figure 3:**
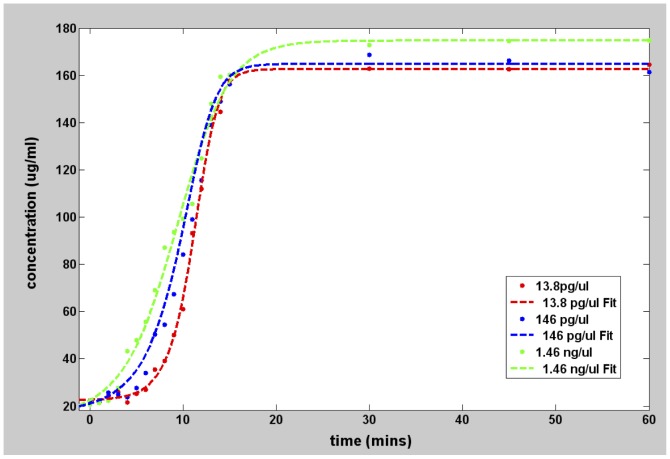
Graphs showing LAMP amplification of various initial concentrations of highly purified genomic O157 target DNA.

Figure 1 shows the concentration versus time for the 0157 and 045 strains, as well as the DH5 alpha negative control strain. Each reading was done in duplicate. Both O157 and O45 were positive to the VT1 and VT2 genes as exhibited by the rapid increase in concentration. For both strains, the concentration of the VT1 and VT2 genes increased exponentially and saturated within 20 minutes. By contrast, the concentration for the negative control DH5 alpha genes showed little to no increase even after 1 hour, in the same manner as the blank target. These observations are consistent with the electrophoresis results shown in Figure 2, in which banding was observed in the O157 and O45 lanes amplified using VT1 and VT2 genes, while no banding was seen for DH5 alpha strain. To understand the influence of the starting concentration, 3 different starting target concentrations of highly purified DNA, namely 13.8 pg/µl, 146 pg/µl and 1.4 ng/µl of the O157 target were examined. Figure 3 shows the data and the corresponding fit for the three dilutions of the target DNA. The three different target concentrations increased exponentially and saturated within 15 minutes, although at different rates.

**Figure 4:**
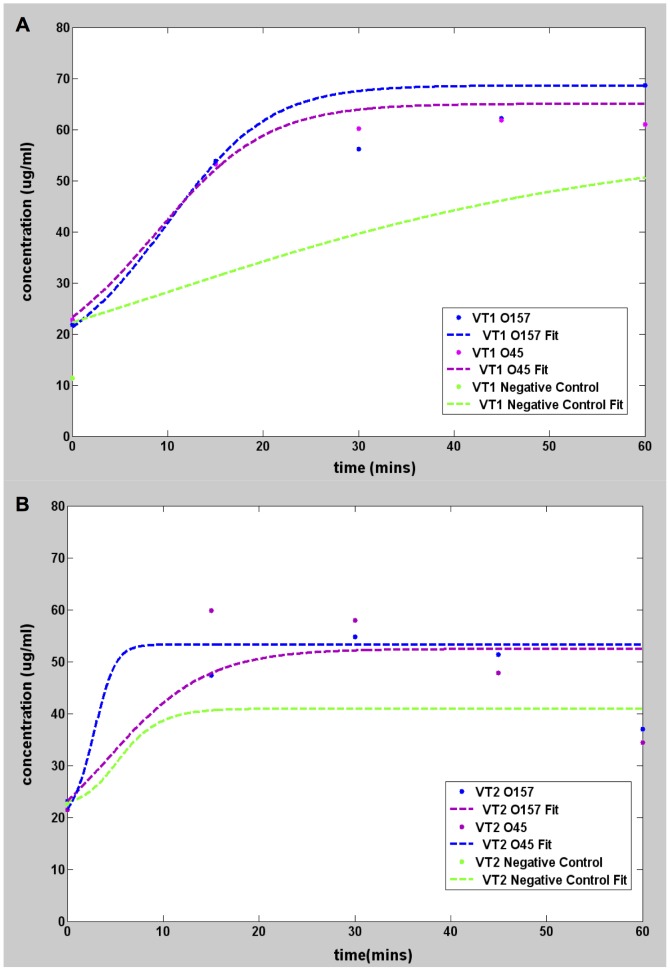
Graphs showing pseudo real time amplification of InstaGene kit purified target DNA. All measurements made using Qubit 2.0. Plots showing DNA amplification using *E. coli* O157, O45 and DH5 alpha (negative control) InstaGene kit purified genomic DNA as target for VT1 gene (Figure 4A) and VT2 gene (Figure 4B).

As with any standard DNA replication, the purity of the target DNA affects the efficiency of amplification. To assess the influence of sample purity, an identical set of experiments using InstaGene kit purified samples were conducted and the result is shown in Figure 4.

## Discussion

### Baseline and saturation reading from Qubit 2.0

From the data, the initial concentrations were in the range 10–30 µg/ml in all samples including the blank with no target DNA. Thus, the initial fluorescence signal must be regarded as the baseline reading from the other elements of the cocktail and has no relation to the actual initial target DNA concentration. Nevertheless, the signal at the onset of saturation (*t>*20 minutes) minus the baseline can be taken as a true measure of the final concentration of the synthesized DNA fragments.

### LAMP mathematical model

As seen from Figure 1, the amplification behavior of the positive samples is in stark contrast with the negative control. The positive samples exhibit a definite ‘turn on’ time after which the concentration precipitously rises. The negative and blank concentrations increase as well but are less abrupt and at significantly lower rates. Thus, one can qualitatively conclude that the O157 and O45 contain VT1 and VT2 genes, whereas the DH5 alpha strain does not.

As valuable as qualitative assessments are, they do not provide information about the amplification rates and the precise threshold time marking exponential replication. These can be obtained using an empirical model of the curves represented by a generalized logistic function. Generalized logistic function or Richard's curve are used in predicting population growth, cancer tumor growth, reaction models, and others in which there are multiple competing factors [17]. In LAMP, there is a competition between the so-called extended cauliflower-like structures and the complementary dumb bell structures in the cycling amplification step. The equation for the generalized logistic function is given by
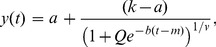
(1)where *y(t)* is the concentration of amplicon at time *t* minutes, *k* is the concentration of amplicon at infinite time and *a* is the lower bound. There are two other variables associated with this equation namely *m* and *b*. In order to impose the condition that the parameter, *m*, reflect the time at maximum growth rate Eq.1 is simplified by setting *Q = ν* [17]. Furthermore, when fitted as free parameters, *Q* and *ν*, are on order unity and weakly influence the other fitting parameters. Thus, for reasons of simplicity, we arbitrarily set these parameters to 1. The data can be fitted using this simplified model so that the parameters *a, k, m*, and *b* can be extracted. Standard numerical techniques are available elsewhere, and in this case, the MATLAB Curve Fit Tool was used to perform the curve fitting.

**Figure 5:**
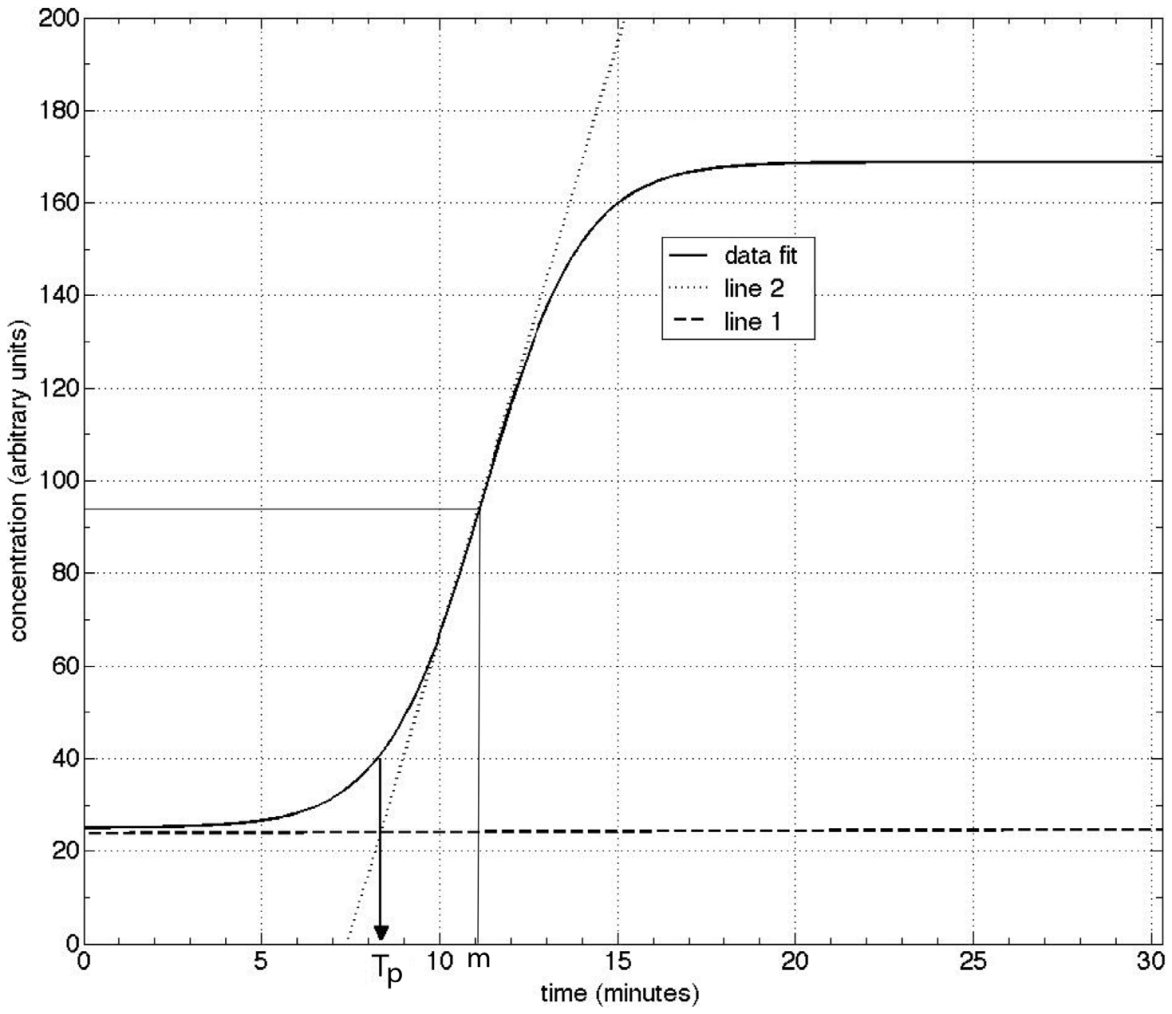
Procedure for determining the time to positive, *T_p_*, as the intersection of the pre-amplification line (line 1) and the linear extrapolation of the amplification growth section (line 2). The data set for O157VT1 is used for this illustration.

Based on these parameters, one can assign a time *T_p_* [5], which is the amplification threshold, analogous to the cycling threshold, *C_T_* in PCR. A unique way to identify this point is assign it as the time that corresponds to the intersection of the initial linear portion of the pre-amplification curve (line 1) and the linearized section of the exponential growth curve (line 2). This is depicted in Figure 5, representing the data for the O157 VT1 set.

The process for obtaining *T_p_* is straightforward, which can be obtained by construction, i.e., manually drawing the lines. Alternatively, one can obtain it analytically as follows. Line 1 is given as
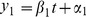
(2)with
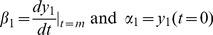
(3)Line 2 is given as,
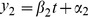
(4)with
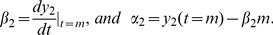
(5)Since, at *t = T_P_*, we obtain
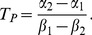
(6)In terms of the fit parameters, we recognize that Eq. (3) and Eq. (4) yield
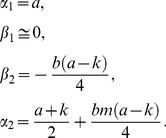
(7)Substitution into Eq. (6) yields a very simple expression for the time to positive,
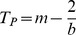
(8)This simple and convenient procedure provides a standardized method for unambiguously extracting *T_p_*. We used Eq. (8) to compute the *T_p_* appearing in Tables 4–6.

**Table 4:** Values of parameters of the Richard's curve fit for graphs of Figure 1 using Equation 1 with Q = v = 1.

Data Set	a (µg/ml)	b (mins^−1^)	k (µg/ml)	m (mins)	R^2^	T_p_ (mins)
O157 VT1	23.84	0.74	163	11.1	0.998	8.39
O157 VT2	26.85	0.77	146.6	10.87	0.996	8.27
O45 VT1	26.03	0.56	127.7	11.49	0.997	7.92
O45 VT2	20.45	0.54	137.2	10.87	0.998	7.18
Neg Cntl DH5 alpha VT1	10.56	0.03	30.9	28.81	0.823	−37.86
Neg Cntl DH5 alpha VT2	16. 73	0.03	37.13	20	0.7769	−46.67
Blank VT1	6.227	0	30	0.54	−1	-inf
Blank VT2	10	0.006	13.11	0	−2	−333.33

Also included are the R^2^ goodness of fit parameter, and the time to positive parameter, *T_p_*, extracted using Equation 6.

**Table 5:** Values of parameters of the Richard's curve fit for graphs of Figure 3 using Equation 1 with Q = v = 1.

Analysis	a (µg/ml)	b (mins^−1^)	k (µg/ml)	m (mins)	R^2^	T_p_ (mins)
O157 VT1 13.8 pg-µl-1	23.84	0.73	163	11.1	0.998	8.36
O157 VT1 146 pg-ul-1	22.03	0.52	165	10.48	0.9975	6.63
O157 VT1 1.46 ng-ul-1	12.39	0.34	174.9	9.05	0.9922	3.16

Also included is the time to positive parameter, *T_p_*, extracted using Equation 6.

**Table 6:** Values of parameters of the Richard's curve fit for less purified target samples extracted using the InstaGene kit.

Analysis	a (µg/ml)	b (mins^−1^)	k (µg/ml)	m (mins)	R^2^	T_p_ (mins)
O157 VT1	10.07	0.2	68.62	2.498	0.801	−7.50
O45 VT1	10	0.19	65	6.062	0.858	−4.46
O157 VT2	10.1	0.1937	52.4	5.123	0.607	−5.20
O45 VT2	17.17	0.1998	53.25	3.948	0.573	−6.06
Neg Cntl DH5 VT1	11.3	0.0355	58.01	20	0.903	−36.34
Neg Cntl DH5 VT2	22.65	0.04	40.9	20	0.54	−30.00

### LAMP using highly purified *E. coli* genomic DNA

The values of the best-fit parameters for each of the curves of Figure 1 are listed in Table 4. The parameter *a*, is the baseline signal and *k* is the saturation signal. As discussed previously, this severely overestimates the starting target concentration. However, *k* can be regarded as a direct indicator of the final concentration. The parameter *m* denotes the time in which the maximum slope of the generalized curve occurs. This number ranges from 10.87 to 11.49 minutes for the positive samples, and significantly longer for the negative control. This parameter is due to the intrinsic latency involved in the creation of sufficiently large numbers of starting dumbbell structures [10] that initiate the cycling amplification step and the subsequent formation of the multi-loop cauliflower-like structures. For the negative samples, the amplification is primarily non-specific, so there is no clear onset for the formation of multiloop structures. The blank samples yielded low values that are essentially noise since the R^2^ parameter is negative, indicating that there is no correlation between the model and the data. The other important parameter is *b* in units of inverse time. It is a measure of the maximum steepness of amplification rate at the exponential growth stage. It can also be interpreted as the reciprocal of the time constant for the reaction, which has to do with the factors that hinder the reaction kinetics, such as steric hindrance or the probability associated with the competition between primer binding. This parameter is the most sensitive between positive and negative samples, and usually varies by more than an order of magnitude. More importantly, the data shows that *T_p_*, given by Eq. (8), can be regarded as an unequivocal indicator of LAMP amplification. Note that the DH5 alpha (negative control) and the blank samples yielded negative *T_p_*. Negative values for *T_p_* are unphysical as they correspond to amplification that occur even *prior* to the start of the LAMP process. Thus, we conclude that negative values for *T_p_* can be regarded as an indicator of a negative LAMP amplification result.

### Effect of starting DNA concentration

**Figure 6:**
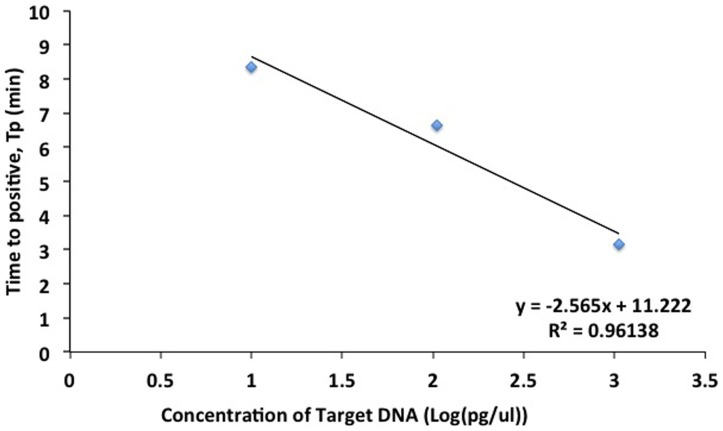
Standard Curve of Time to positive *T_p_* versus log of concentration (in pg-µl^−1^).

The best-fit parameters for the curves of Figure 3 are summarized in Table 5. The tenfold dilution series, which spans pg-µl^−1^ to ng-µl^−1^ range, shows a highly linear relationship between with *T_p_* as shown in Figure 6. Also seen from Table 5, *m* is inversely related to the initial DNA concentration. Recalling that *m* represents the time for maximum rate of amplification, this is expected since the probability for the primers to bind to one of the many target DNA regions of the genomic DNA is proportional to the initial target concentration. Higher initial target concentration leads to rapid creation of the starting (dumbbell) structure, and consequently, the maximum amplification rate occurs earlier.

Parameter *b* can be regarded as the maximum rate of amplification of the target. Mathematically, it can be defined as the change in concentration over unit time, which graphically is the slope of the Richard's curve at its steepest part or at time *t = m*. As Table 5 shows, the parameter *b* is inversely related to the concentration, i.e., the fastest rate is observed for 13.8 pg/µl and the lowest for 1.4 ng/µl. Although, the exact mechanism has to be rigorously established, we hypothesize that after *m* minutes have elapsed, there is enhanced competition between the genomic DNA and the amplicons for the primers. If the primers were to bind to the genomic DNA the starting dumbbell structure will be produced, whereas if they were to bind to an amplicon (either a starting-dumbbell structure or a cauliflower structure), then amplification will occur. Thus, in the case of high target concentration, the overwhelming amount of genomic DNA relative to the amplicons will cause the primers to preferentially bind to the genomic DNA, than to the amplicons, thereby reducing the rate of amplification. Conversely, in the case of a lower concentration of genomic DNA, the number of the amplicons produced after time *m* may become comparable to the amount of genomic DNA targets so that the probability of the primers binding to the amplicons becomes comparable or even higher than the probability of the primers binding to the genomic DNA. This could explain why the amplification rate observed in our results is highest at the lowest concentration.

### LAMP reaction using InstaGene kit extracted *E. coli* genomic DNA

Following the same protocols as before, LAMP was performed on the less (Instagene kit) purified samples. The initial concentration was in the range of 1 µg/µL as was used in the previous samples. The amplification curves are shown in Figure 4, and, the fitting parameters are summarized in Table 6. The main difference with the highly purified samples is that the *b* parameter representing the maximum rate of amplification is significantly lower in the less purified samples. With such low sensitivity it is unclear if the DNA synthesis is due to amplification of the target or to non-specific binding, which obscures the interpretation of LAMP. Fortunately, this conundrum can be resolved by calculating *T_p_*. Substitution of *b* and *m* from the fit into Eq. (8) yields negative values for *T_p_*. Having established earlier (see Table 5) that negative *T_p_* was an indicator of a null result for negative-control and blank sample, it can be concluded that VT1 and VT2 genes were not synthesized in the less purified samples. PCR amplifies a single strand of the target DNA stepwise during thermal cycles, whereas LAMP does so continuously using a double stranded DNA template and strand displacement based DNA synthesis. Thus, the LAMP method of DNA amplification may be very sensitive to the additional substances present in less purified samples that interfere with the DNA synthesis process. Hence it is not surprising that Instagene purified samples work in PCR but not in LAMP. Still, other researchers report that LAMP could be less affected by inhibitory substances in the clinical sample than PCR and that the purification step can be omitted [18]. In fact, it has been recently reported that *E. coli* can be detected by LAMP directly from urine samples without DNA extraction [19]. That these results are at odds with ours may be understood by noting that their study targeted the *E.coli* malB gene, which is a conserved gene across diverse lineages of *E. coli*. Because it is non-discriminatory, their assay is extremely sensitive and thus more robust against impurities. But our experiment targets very specific vero-toxin markers, and it is perhaps this stringency that makes LAMP sensitive to target purity in this case.

## Conclusions

In conclusion, a model using a simplified generalized logistic function (Richard's curve) is proposed to quantify the LAMP amplification process. The technique was exemplified in identifying vero-toxin producing O157 and O45 *E. coli* stains from highly purified samples, as well as discriminating the non-VT strain and assessing the influence of impurities. The model yields several parameters that uniquely describe the amplification curve as a function of time and from which, the time to positive, *T_p_*, was extracted. In general, the modeling offers several advantages. First, it is a compact way to characterize individual concentration growth curves using only 4 parameters. This allows easy analysis, transmission and archival of data, which will become even more important as LAMP screening becomes more prevalent. Second, the proposed model can be used as a universal standard for comparing LAMP amplification. By using a heuristic model, researchers can meaningfully compare results from different techniques for measuring the concentration (e.g. fluorescence, optical absorbance, turbidity). Standard curve fitting packages are available e.g., MatLab which is straightforward to set up. Third, the quantification removes human bias in interpreting the results so that it becomes possible to perform high throughput and automated data screening. Finally, a universal standard method for expressing LAMP data will facilitate statistical inferences involving large population sizes.

## Supporting Information

Figure S1 **Calibration plot for Qubit 2.0.**
(TIFF)
