# Supplementary figures and images for: An Empirical Approach for Quantifying Loop-Mediated Isothermal Amplification (LAMP) Using Escherichia coli as a Model System
Source: PLoS One. 2014 Jun 30;9(6):e100596. doi: 10.1371/journal.pone.0100596 (PMC4076223; doi:10.1371/journal.pone.0100596)

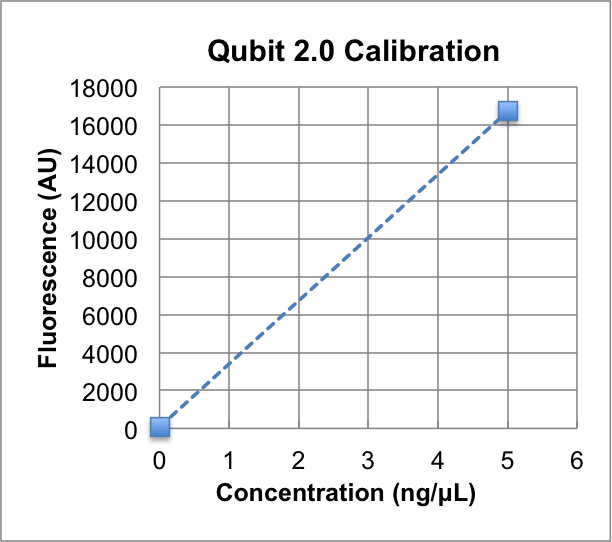

Supplement: Figure S1 — Calibration plot for Qubit 2.0. (TIFF) [file pone.0100596.s001.tiff]
